
JOURNAL INFORMATION
==============================
NLM Title Abbreviation: Buffalo Med J Mon Rev Med Surg Sci
NLM Journal ID: 0004276

The Buffalo Medical Journal and Monthly Review of Medical and Surgical Science

Publisher: Biomedical Journal Digitization Project

ARTICLE INFORMATION
==============================
PMCID: PMC8939054
Article version: 1
Subjects: Editorial Department

A Practical Treatise on the Diseases of Children

Print publication date: 1858 Feb
Volume: 13
Issue: 9
First page: 575
Last page: 576
License: This work is free of known copyright restrictions. For more information, please see PMC Back Issue Digitization.
License URL: https://creativecommons.org/publicdomain/mark/1.0/

==============================

of one of our most able collaborators. We were anxious to present to our readers in the present number, the article on microscopic examination of blood stains, by Robin, which we think is a paper of great practical utility. Several books stand over to be noticed in our next number. F.

---

*A Practical Treatise on the Diseases of Children.* By J. FORSYTH MEIGS M. D., Fellow of the College of Physicians of Philadelphia; Member of the American Philosophical Society, and of the Academy of Natural Sciences of Philadelphia. Third Edition, carefully Revised. Philadelphia: Lindsay & Blakiston. 1858.

This admirable text-book on the diseases of children, has now become a standard work. The labors of the author have added much entirely new matter to the first edition, which formed one of a series entitled "The Medical Practitioner's and Student's Library." The third edition has been carefully revised, and much new matter incorporated into the text, though the author has been prevented from making additions of chapters on subjects not touched upon, which he had proposed to do in the present edition. We hope that this promise will be fulfilled, however, in another edition. F.

---

*American Medical Monthly.*—The proprietors of this Journal have enlarged the volume by an addition of sixteen pages to each number. We are glad to see this evidence of prosperity, and can bear testimony that it is well deserved. The Monthly has always been conducted with high-minded spirit and candor on the part of the editors, and its pages have been enriched by many valuable contributions to science. F.

---

*Editorial Change.*—With the last number of the twelfth volume of the Charleston Medical Journal, Dr. C. Happolt retires from its editorial management and proprietorship. Dr. Happolt retires with our sincere wishes for his future welfare and our respect for the able manner in which he has conducted his journal. The new editor, Dr. J. Dickson Bruns, is already favorably known to the profession by some important and interesting contributions, and will no doubt sustain himself well in his new position. F.

*The North American Medico-Chirurgical Review.*—The size of this sterling periodical has been enlarged with the cammencement of the second volume, making now one hundred and ninety-two pages to each number. this journal was commenced in Philadelphia on the first of January, 1857, by Profs. Gross and Richardson, the former editors of the Louisville Review; the sound and able articles and reviews which have appeared in its pages for the past year have made it one of the most agreeable and profitable of our exchanges, and have rendered its success sufficiently flattering to induce the publishers to enlarge it to its present dimensions. The appearance which it now presents in point of typography and style is highly creditable, and the editorial management is such as to make it rank with the best Journals of any country. F.

---

*Consolidation.*—The Western Lancet and the Cincinnati Medical Observer, have been consolidated under the name of the Cincinnati Lancet & Observer. Some of the circumstances which induced this change are mentioned in the last number of the Lancet. The want of a sufficiently energetic corps of colaborators, and the desire to diminish the number of of rival factions and interests, and unite the talent of her writers, are mentioned as the principal causes which have made this step advisable. The consolidated number looks well. F.

---

*A New Medical School at Nashville.*—We learn that the brilliant success of the medical department of the University has prompted a new enterprise in the way of a Medical School at Nashville. We understand that it is to be under the patronage of the Methodist denomination.—*Cincinnati Lancet & Observer.*

---

*New Sydenham Society.*—An effort has been made to organize another Sydenham Society, for the purpose of republishing modern works and papers, and more especially those of a practical class. A second meeting will soon be held, and the matter definitely settled.—*Western Lancet.*

---

*The University of London and Homœopathy.*—At a late examination for the degree of M. B. of the University of London, a candidate, who was known to profess and practice homœopathy, was rejected.—*Ib.*
